# Gut Dysbiosis and Kidney Diseases

**DOI:** 10.3389/fmed.2022.829349

**Published:** 2022-03-03

**Authors:** Chujin Cao, Han Zhu, Ying Yao, Rui Zeng

**Affiliations:** ^1^Division of Nephrology, Tongji Hospital, Tongji Medical College, Huazhong University of Science and Technology, Wuhan, China; ^2^Division of Nutrition, Tongji Hospital, Tongji Medical College, Huazhong University of Science and Technology, Wuhan, China

**Keywords:** gut microbiota, chronic kidney disease, metabolic disorders, probiotics, FMT

## Abstract

Gut dysbiosis is defined as disorders of gut microbiota and loss of barrier integrity, which are ubiquitous on pathological conditions and associated with the development of various diseases. Kidney diseases are accompanied with gut dysbiosis and metabolic disorders, which in turn contribute to the pathogenesis and progression of kidney diseases. Microbial alterations trigger production of harmful metabolites such as uremic toxins and a decrease in the number of beneficial ones such as SCFAs, which is the major mechanism of gut dysbiosis on kidney diseases according to current studies. In addition, the activation of immune responses and mitochondrial dysfunction by gut dysbiosis, also lead to the development of kidney diseases. Based on the molecular mechanisms, modification of gut dysbiosis via probiotics, prebiotics and synbiotics is a potential approach to slow kidney disease progression. Fecal microbiota transplantation (FMT) and genetic manipulation of the gut microbiota are also promising choices. However, the clinical use of probiotics in kidney disease is not supported by the current clinical evidence. Further studies are necessary to explore the causal relationships of gut dysbiosis and kidney diseases, the efficiency and safety of therapeutic strategies targeting gut-kidney axis.

## Introduction

Kidney disease is a major public health concern worldwide and is associated with the high morbidity and mortality, with the various pathogenic mechanisms related to immune responses, oxidative stress, inflammation, metabolic disturbance and so on (1–5). However, lack of effective strategies for the prevention and management of renal disease is still an important issue urgently needed to be addressed. For example, how to slow the progression of chronic kidney disease (CKD) is tough due to the current therapeutic strategies just aiming at the decrease on proteinuria and the control of elevated blood pressure (6). Thus, novel targets and treatments become the hot spot in mitigating the growing burden of kidney diseases.

Human gastrointestinal (GI) tract contains more than 100 trillion microorganisms, which forms a microbial community with abundance and diversity (7). Bacteria are one of microorganisms and classified on the level of phyla, classes, orders, families, genera, and species. The dominant bacteria on phylum level are Firmicutes, Bacteroidetes, Actinobacteria, Proteobacteria, Fusobacteria, and Verrucomicrobia. Among these, Firmicutes and Bacteroidetes predominate and represent 90% of the bacteria in gut (8). Gut microbiota composition is highly variable and maintain dynamic balance in the physiologic condition. However, gut microbiota variations tend to be one of the important implications in intestinal and extra-intestinal disorders. These variations are defined as gut dysbiosis, which includes disorders of gut microbiota composition and loss of intestinal barrier integrity (9–11). For examples, pathogenic bacteria were increased, and uremia toxin levels were elevated under the condition of CKD. The disruption of intestinal epithelium integrity enhances harmful gut-derived metabolites into circulation, in turn, aggravating kidney injury (9). Gut dysbiosis have dual identities as not only the contributing factors to kidney disease progression, but also increasing the risk for various kidney diseases especially in individuals with genetic predisposition (12–16). Therefore, understanding the complex relationships between gut dysbiosis and kidney diseases may provide us with potential therapeutic strategies.

Recent technological advances in omics have arisen our knowledge of gut microbiota. The next generation sequencing technologies, including 16S ribosomal RNA (rRNA) or DNA and metagenomics sequencing analysis identified a large number of gut microorganisms and differential species between healthy subjects and diseased individuals (11). In addition, metabolomics sequencing has become the most common technique to explore the alterations of gut metabolites, while single-cell sequencing is widely used to dig out the specific molecular mechanisms (17, 18).

In this review, we aimed to describe the manifestation of gut dysbiosis in kidney diseases according to numerous clinical studies and animal experiments. Furthermore, we discussed the molecular mechanisms of gut dysbiosis contributing to the pathogenesis and progression of kidney diseases. Finally, we focused on the potential therapeutic strategies targeting gut dysbiosis on kidney disease treatments.

## Gut Dysbiosis in Kidney Diseases

16S rRNA or DNA sequencing is the common method to evaluate microbial diversity and identify differential microbes in patients compared with healthy control (HC) subjects. Numerous observational clinical studies demonstrated that patients with CKD, in general, exhibited a prominent reduction in abundance and diversity of fecal microbiota (19–23). At the phylum level, the abundance of Actinobacteria and Firmicutes was reduced in the CKD groups compared with the HC group, while that of Verrucomicrobia Fusobacteria and Proteobacteria was increased (19, 24). Moreover, there were some differences among the altered gut microbiota at the genus and species level in individuals with CKD (23). The individual differences caused by genetic and environmental factors might account for the variety and inconsistence of results. Beyond that, the severity and etiology of CKD in clinical stages and pathological classifications also led to the different manifestations of gut dysbiosis in the enrolled-in patients. For patients with CKD of 3–5 stages, Lactobacillus, Clostridium IV, Paraprevotella, Clostridium sensu stricto, Desulfovibrio, and Alloprevotella were abundant in the fecal samples, while Akkermansia and Parasutterella were enriched in those of HC subjects (19, 23). Another study indicated that fecal microbiota had significantly higher abundance of Citrobacter, Coprobacillus and lower abundance of Prevotella spp., Faecalibacterium prausnitzii, Roseburia spp. in patients with CKD of 3b-4 stages compared to HC subjects (20). A group of microbial species were enriched in patients with end-stage renal disease (ESRD), including Eggerthella lenta, Flavonifractor spp, Alistipes spp, Ruminococcus spp and Fusobacterium spp, while several species were depleted, including Prevotella spp, Clostridium spp, Roseburia spp, Faecalibacterium prausnitzii and Eubacterium rectale (21). CKD patients with dietary protein restriction showed an increase in intestinal Escherichia, Shigella, and Klebsiella and a decrease in Blautia (25). When accompanied with idiopathic nephrotic syndrome (INS), patients with CKD held decreased Megamonas, Megasphaera, Akkermansia, Lachnospira, Roseburia and Fusobacterium compared with CKD patients without INS (24). The significant gut microbiota disorders also varied among kidney diseases with distinct pathological patterns. For examples, Escherichia-Shigella and unclassified Defluviitaleaceae were increased in IgA nephropathy (IgAN) compared with HC, whereas Roseburia, unclassified Lachnospiraceae, sensu stricto Clostridium, and Fusobacterium were decreased. Escherichia-Shigella, Peptostreptococcaceae, Streptococcus, and unclassified Enterobacteriaceae were increased in MN, whereas Lachnospira, unclassified Lachnospiraceae, sensu stricto Clostridium, and Veillonella were decreased. When comparing IgAN with MN, Megasphaera and Bilophila were increased, while Megamonas, Veillonella, Klebsiella, and Streptococcus were decreased in IgAN (26). These results described the landscape of intestinal flora in kidney diseases. Identification of differential bacteria is the first step to dig out the potential gut-associated target on treatments of kidney diseases.

Whether the altered gut microbiota is correlated with clinical characteristics of kidney diseases is of much importance, which determines the significance of gut dysbiosis in the diagnosis and treatments of renal diseases. The correlation analysis demonstrated that the butyrate-producing bacteria, such as Faecalibacterium and Prevotella, were negatively related to the serum level of C-reactive protein (CRP) and Cystatin C (CysC) in ESRD patients (27). In addition, Akkermansia was negatively correlated with inflammatory indicators like interleukin-10 production in CKD patients, suggesting Akkermansia might be a novel biomarker of CKD (19, 22). In diabetic nephropathy (DN), the genus Escherichia-Shigella and the genus Prevotella 9 levels in feces might be used to distinguish DN from diabetes mellitus, which may contribute to the early diagnosis of DN (28). Further, Anaerosporobacter might be a harmful factor and Blautia might be a protective factor in DN due to the positive and negative correlation with 24-h urinary protein content, respectively (29). In individuals with IgAN, Prevotella was positively correlated with serum albumin level, while Klebsiella, Citrobacter, and Fusobacterium were negatively correlated. Furthermore, Bilophila was identified positively correlating with crescents in the Oxford classification of IgAN. Similarly, the negative correlation was existed between Escherichia-Shigella and proteinuria in individuals with MN. Bacteroides and Klebsiella exhibited positive correlation with different MN stages. Thus, the alterations of gut microbiota might also serve as implications of clinical and pathological severity of IgAN and MN (26). These observations indicate that gut microbiota can be used as a novel kind of biomarker for kidney diseases in diagnosis and prognosis. However, large-scale prospective clinical studies are needed to further confirm the reliability of setting the microbial biomarkers.

Dialysis, as one of the effective therapeutic strategies for patients with ESRD, has been showed to improve gut dysbiosis. In patients treated with hemodialysis (HD), the numbers of fecal Bifidobacterial and Lactobacillus acidophilus were higher, while Escherichia coli and Enterococcus faecalis numbers were lower than those of patients without HD treatment. Compared with ordinary hemodialysis, combination treatment with hemoperfusion dialysis could further improve gut microbial disorders with higher number of fecal Lactobacillus acidophilus and lower number of Escherichia coli (30).

In addition, individuals with peritoneal dialysis (PD) presented with different changes of gut microbiota compared with patients with HD. Fecal Proteobacteria was decreased in HD patients, while increased in PD patients (31). It also showed that Bacteroidetes were significantly deceased in HD patients compared with pre-dialysis patients. HD was observed reversing the dysbiosis in pre-dialysis patients (32). Further analysis demonstrated that Bacteroides and Phascolarctobacterium were related to cardiovascular mortality in patients with dialysis (32). The number of fecal Dorea, Clostridium, and SMB53 were associated with peritonitis in PD patients, suggesting gut dysbiosis predicts prognosis of patient with PD (32). These findings imply that monitoring gut dysbiosis in individuals with CKD is necessary for providing specific treatment suggestions.

To determine the exact causations between gut dysbiosis and kidney diseases, renal-injured germ-free or antibiotic-treated rodents were transplanted with microbiota from CKD patients or healthy controls. The results showed that CKD patient-derived microbiota induced higher levels of serum uremic toxins and aggravated renal fibrosis as well as oxidative stress more than that from healthy controls (21). The pathogenic role of gut microbiota from CKD patients was confirmed by fecal microbiota transplantation. Moreover, microbiome changes induced by CKD might be exacerbated in the process of renal transplantation with immunosuppression (33). A study on renal transplantation patients indicated that spousal pairs with similar microbial composition had better 6-month allograft function and lower morbidity of post-transplantation infection. Correlation analysis showed that the pre-transplantation microbial similarity in donors and recipients hold a fantastic accuracy in the prediction of the estimated glomerular filtration rate (eGFR) at 6-months post transplantation (21, 34). These data suggest that gut dysbiosis is not only an intestinal manifestation in kidney diseases but also an important factor that leads to disease progression. Thus, it is necessary to explore the molecular mechanisms of gut dysbiosis on aggravating renal diseases and treat patients with kidney diseases by modifying gut microbiota.

## Molecular Mechanisms of Gut Dysbiosis on Kidney Diseases

It is obvious that kidney diseases are accompanied by gut dysbiosis, which is a promotive factor involving in the progression of diseases. However, as the manifestations appeared in distal organs, how gut dysbiosis impacts on kidney disease progression becomes an urgent demand. The development of metabolomics sequencing helps us to explore the link underlining molecular basis of gut-kidney crosstalk. Multi-omics combined analysis showed that altered gut microbial species linked to intestinal, circulating and renal metabolites, including uremic toxins, short-chain fatty acids and trimethylamine (TMA) (21, 35–39).

### Uremic Toxins

Excessive production of uremic toxins is a consequence of gut microbiota alteration, including indoxyl sulfate (IS) and *p*-cresyl sulfate (PCS) (11). Mice with depleted gut microbiota were transplanted with microbiota from CKD patients, finally detected higher levels of serum uremic toxins, suggesting that altered gut microbiota might aggravate CKD progression by increasing the production of uremic toxins. Moreover, two of the species, Eggerthella lenta and Fusobacterium nucleatum, were identified to increase uremic toxins productions and deteriorate CKD progression (21). Protein post-translational modifications in bacteria have been detected and characterized associated with nitrogen metabolism (40, 41). Based on the fact, Lior Lobel et al. found that microbial tryptophanase activity was posttranslationally modified by a high sulfur amino acid-containing diet, which reduced the activity of uremic toxin production and ameliorated CKD progression in mice (35). However, a study showed that serum uremic toxins levels were upregulated in patients with different stages of CKD, while remained the same in fecal and urine samples (36). Therefore, an increase in these toxins cannot be entirely explained by increase in bacterial generation in gut. The decrease of fractional clearance due to the renal function decline in CKD patients appeared to increase uremic toxins levels in serum. Meanwhile, the disruption of intestinal epithelial barriers was also one of the important causes to the influx of uremic toxins into circulation (36). Nevertheless, daily medications for CKD patients also interferes the metabolism of these uremic toxins. For examples, two-week canagliflozin treatment for adenine-induced renal failure in mice did not prevent the impaired renal function. However, it significantly reduced the plasma levels of IS and PCS, meanwhile, reversed gut dysbiosis (42). These data suggest that increased production of uremic toxins caused by gut dysbiosis might contribute to CKD progression despite the causes for the increase of these toxins are various.

### Short Chain Fatty Acids

SCFAs are generated from indigestible dietary carbohydrate fibers by the intestinal microbiota, mainly including acetate, propionate and butyrate (43). Depletion of gut microbiota using antibiotics in diabetic rats markedly reduced serum acetate levels. As expected, fecal microbiota transplantation from the healthy donor also effectively decreased serum acetate levels. These animal experiments indicate tight correlations between gut dysbiosis and SCFA level alterations. Furthermore, SCFAs were showed to play a critical role in slowing CKD progression. Butyrate was almost three times lower in serum of CKD patients than that of HC. A negative correlation was observed between butyrate level and renal function, which was improved by supplementation with extra butyrate (44). Our previous study confirmed that oral administration of the probiotics *Lactobacillus casei Zhang* (Lac.z) increased SCFA levels in serum and kidney, which prevented acute kidney injury (AKI) and delayed renal fibrosis progression (45). Thus, regulation of gut dysbiosis, manifested as inducing an increase in SCFA-producing bacteria, may lead to higher levels of SCFAs in circulation and kidney, further reducing tubular cell injury and tubulointerstitial fibrosis.

### TMA and TMAO

Nutrient precursors, abundant in red meat and a Western diet, such as phospatidylcholine choline and carnitine, were used by gut microbiota as a carbon fuel source to generate TMA as a waste product, which was subsequently absorbed into circulation and converted within the liver to trimethylamine N-oxide (TMAO) (46, 47). Dietary supplementation of TMAO significantly aggravated the impairment of renal function and the progression of renal fibrosis, reflecting as the decline of eGFR, and the increase of CysC, albumin/creatinine and renal tubulointerstitial fibrosis score (37). Transplantation microbiota from CKD patients to rats were confirmed to accelerate CKD progression by increasing TMAO production (44). The specific medication like iodomethylcholine, a gut microbiota-dependent choline TMA-lyase mechanism-based inhibitor, reversed choline and adenine diet-induced gut microbial community composition related to TMA and markedly suppressed TMA generation, subsequently TMAO level, thus alleviating renal functional impairment (37, 48). In addition, ranitidine and finasteride also inhibited the synthesis and metabolism of TMAO to exhibit potential protective effects on CKD and cardiovascular complications (49). These data show that TMA and TMAO production is dependent on gut microbiota and become a potential target to attenuate renal disease progression.

A rigorous study was performed that patients with CKD were highly selected with strict inclusion criteria to eliminate the influence of the confounding factors on the microbial composition changes. The results showed that gut Lactobacillaceae family displayed a stepwise change in relative richness responding to renal insufficiency from mild, moderate to severe group. In addition, the uremic toxin pathway was associated with microbial changes in CKD patients (50). Despite that, the question remains to be answered whether these metabolite alterations are the results of gut dysbiosis and trigger kidney disease development or they just come with the occurrence and progression of diseases. More designed studies, larger-size samples and multidimensional analysis are needed to clarify the causal or concomitant relationships among gut dysbiosis, metabolite changes and kidney disease progression.

Gut is abundant in what called gut-associated lymphoid tissue, suggesting gut dysbiosis and disease-related metabolite alterations may impact on kidney diseases by activating immune responses. TLR7-dependent translocation of Lactobacillus reuteri was confirmed to induce the increase in plasmacytoid dendritic cells (pDCs) and interferon signaling, which worsened autoimmune responses in systemic lupus erythematosus (SLE). While dietary resistant starch suppressed the abundance and translocation of Lactobacillus reuteri, resulting in a decrease in pDCs, which were benefit to patients with SLE (51). It also mitigated inflammation via expansion of Tregs to slow CKD progression (52). However, another study showed that treatment with a mixture of five Lactobacillus strains skewed the Treg-Th17 balance toward a Treg-dominant phenotype in the kidney of MRL/lpr mice, suggesting that gut microbiota protected against lupus nephritis by expanding Tregs (53). Although the percentage of Tregs was not different between CKD and control mice, pro-inflammatory/resident macrophages increased in the colon of CKD mice. Moreover, probiotics could restore the high percentage of pathogenic macrophages and increased regulatory dendritic cells in the colon. These immune-related changes suppressed systemic inflammation and kidney fibrosis (54). Depletion of gut microbiota with broad-spectrum antibiotics in mice induced lower levels of F4/80 and chemokine receptors CX3CR1 and CCR2 in renal resident macrophages and bone marrow monocytes compared with control mice. In addition, the migratory capacity of monocytes was decreased in gut bacteria-depleted mice. These inflammatory changes with gut microbiota depletion protected mice against renal ischemia reperfusion injury (55). Our previous study also confirmed that the reno-protective role of the probiotics Lac.z was dependent on the decrease in renal macrophages, including inflammatory subset, intermediate-stage subset, and proliferating subset. Further, we used CCR2-KO mice to confirm that the protection was partially dependent on inhibition of CCR2^+^ macrophages. In addition, Lac.z-induced increase in SCFA levels contributed to the reno-protective role by interaction with the classical SCFA-related receptors, GPR43 and GPR109a expressed on neutrophils, and macrophages, which resulted in the anti-inflammatory effect (45). In mice with crescentic glomerulonephritis, gut-derived Th17 cells were confirmed to migrate into kidney via S1PR1-dependent CCL20/CCR6 axis and trigger autoimmune responses in kidney. Depletion of intestinal Th17 cells in germ-free and antibiotic-treated mice ameliorated autoimmune-mediated renal disease, whereas expansion of these cells on condition of Citrobacter rodentium infection exacerbated pathology (56, 57). Therefore, gut dysbiosis may deteriorate kidney diseases by interacting with immune cells.

**Figure 1:**
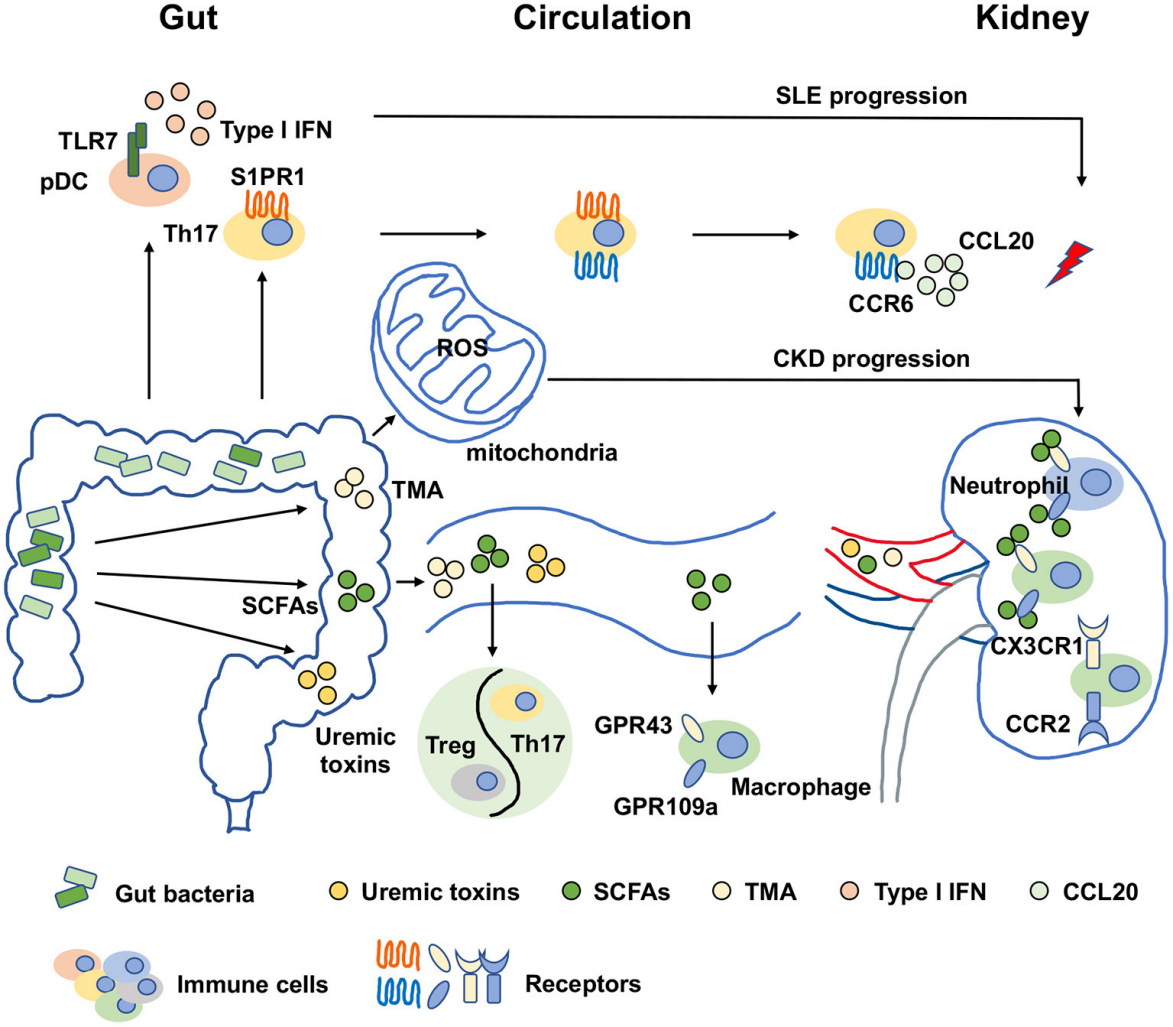
Molecular mechanisms of gut dysbiosis on kidney diseases. The molecular mechanisms of gut dysbiosis on kidney diseases were major focused on two aspects. One was that gut dysbiosis-induced metabolic disorders, manifested as an increase in harmful metabolites such as TMA and uremic toxins and a decrease in beneficial metabolites such as SCFAs, which might directly promote the pathogenesis and progression of kidney diseases. The other was related to immune response activation. Gut dysbiosis and metabolic disorders could expand or activate immune cells by binding specific receptors. For examples, SCFAs regulated macrophages in kidney in a GPR43 and GPR109a-dependent manners, and the activation of pDCs were dependent on TLR7 to induce IFN production on the condition of gut dysbiosis. In addition, another novel perspective has arisen recently that gut dysbiosis-induced the increase of ROS production resulted in mitochondrial dysfunction, which was also important for CKD progression. SCFAs, short chain fatty acids; TMA, trimethylamine; pDC, plasmacytoid dendritic cell; Treg, regulatory T cell; Th17, type 17 of T helper cell; IFN, interferon; ROS, reactive oxygen species; TLR7, toll-like receptor 7; S1PR1, sphingosine-1-phosphate receptor 1; CCR6, chemokine receptor 6; CCL20, C-C chemokine ligand 20; CX3CR1, CX3C chemokine receptor 1; CCR2, C-C chemokine receptor 2; GPR43, G-protein coupled receptor 43; GPR109a, G-protein coupled receptor 109a; SLE, systemic lupus erythematosus; CKD, chronic kidney disease.

The metabolic disturbances in CKD patients, such as oxidative stress and inflammation, promote vascular damage, which is potentially linked to the mitochondrial dysfunction in the enterocytes. Many factors have been reported to control mitochondrial function such as peroxisome proliferator activated receptor gamma coactivator 1 alpha (PGC-1α), which increases the mitochondrial electron transport chain and DNA copy numbers (58). The disturbances in the biogenesis, bioenergetics, morphology and degradation of mitochondria were reported along with PGC-1α expression reduction in CKD (59, 60). Bacterial infections were developed in parallel with mitochondrial dysfunction, which hints that the relationship of gut dysbiosis and mitochondrial dysfunction (61, 62). Gut dysbiosis-induced bacterial metabolites like hydrogen sulfide, uremic toxins, bile acids and lipopolysaccharide interfered with mitochondrial dysfunction by increasing ROS production. Thus, the gut microbiota-mitochondria axis is also important for CKD treatment (63). However, the profound mechanisms are needed to be further explored. In summary, gut dysbiosis affects kidney diseases in the manner of metabolite alterations, immune response activation and mitochondrial dysfunction, which is shown in Figure 1.

## Therapeutic Strategies Targeting Gut Dysbiosis on Kidney Diseases

Based on the data from 16S rRNA or DNA sequencing and metagenomic sequencing, microbial dysbiosis can be corrected by exogenous probiotics supplementation to prevent kidney diseases. Among that, Lactobacillales and Bifidobacteri are the most common probiotics. In patients with stable CKD stage 3a, compared to placebo group, probiotics treatment increased fecal Lactobacillales and Bifidobacteria concentrations, induced significant improvements of CRP, iron status, intact parathyroid hormone (iPTH) and β2-microglobulin with possible beneficial effects on cardiovascular outcomes, particularly when the early treatment was performed (64). We found that Lac.z intervention on individuals with CKD in stage 3–5 resulted in a lower serum CysC. Supplementation with Lactobacillus restored the abundance of SCFA-producing bacteria, leading to increased SCFA levels in gut, circulation, and kidney (45, 65). Another group gavaged the CKD rats with Bifidobacterium animalis A6 and found that the abundances of two toxin-driving species, E. lenta and Fusobacterium spp were significantly decreased even though the overall microbial composition was not significantly altered. Meanwhile, serum levels uraemic toxins, creatinine and urea were decreased and renal fibrosis and glomerulosclerosis were reduced (21). In hyperuricemic animals, probiotics containing uricolytic bacteria lowed serum uric acid with benefit on blood pressure and renal disease (66). In obese-induced kidney injury, Lactobacillus paracasei HII01 supplementation alleviated kidney inflammation, endoplasmic reticulum stress, and apoptosis, resulting in improved kidney function. In addition, the probiotics also induced the attenuation of hyperlipidemia, systemic inflammation, and insulin resistance, along with gut dysbiosis improvement (67). Moreover, a mixture of five Lactobacillus strains (Lactobacillus oris, Lactobacillus rhamnosus, Lactobacillus reuteri, Lactobacillus johnsonii, and Lactobacillus gasseri) contributed to decreased IL-6 and increased IL-10 production with an anti-inflammatory environment in the gut and circulation. Moreover, Lactobacillus treatment increased IL-10 and decreased immune deposit in the kidney of MRL/lpr mice (53). However, the safety and dosage of probiotics need to be strictly controlled due to the liveness of probiotics. Despite that the efficiency of probiotics administration on various renal diseases was confirmed by numerous animal experiments, the placebo-controlled, two-blind, and randomized clinical trials are in serious lack, and current studies show few direct efficiencies such as preventing eGFR decline or serum creatinine increase and urea, which are the stumbling block of applications of probiotics (68). The studies on the effects of probiotics on CKD were listed at Table 1.

**Table 1:** The studies on the effects of probiotics on CKD.

**Probiotics**	**Methods**	**Effects**	**References**
Bifidobacte-rium adolescentis or B. longum	The animals were treated with the probiotics and subjected to kidney IRI.	The probiotic treatment protects mice from IRI-induced CKD: lowered serum levels of creatinine and urea lowered levels of cytokines and chemokines in serum increased acetate production.	(69)
L. casei Zhang (Lac.z) or L. acidophilus (Lact)	The mice were treated with the probiotics for 4 weeks and then subjected to kidney IRI.	The probiotics protect mice from IRI-induced CKD and the effects of Lac.z are more outstanding: reduced serum creatinine and BUN alleviated renal fibrosis improved gut dysbiosis increase the levels of SCFAs and nicotinamide regulated immune responses.	(45)
Lactobacillus paracasei and Lactobacillus plantarum	The mice were treated with low dosage or high dosage of probiotics for 6 weeks and then fed adenine to induce CKD.	The mixed lactic acid strains protect mice from adenine-induced CKD: improved the kidney function reduced kidney injury and fibrotic-related proteins decreased oxidative stress and proinflammatory reactions elevated immune responses in the kidney reversed gut dysbiosis and restored the abundance of commensal bacteria improved intestinal barrier integrity.	(65)
Bifobacterium bifidum A218, Bifidobacterium catenulatum A302, Bifidobacterium longum A101, and Lactobacillus plantarum A87	A randomised, double-blind, placebo-controlled trial: PD patients in the intervention group received one capsule of probiotics daily for six months. The placebo group received similar capsules with maltodextrin for the same duration.	The mixed probiotics are beneficial to PD patients: significantly reduced the serum levels of endotoxin and proinflammatory cytokines (TNF-α, IL-6 and IL-5) increased the serum levels of anti-inflammatory cytokine (IL-10) preserved residual renal function.	(70)
Synbiotics	A randomized, double-blind, placebo-controlled, crossover trial: synbiotic therapy over 6 weeks (4-week washout)	The synbiotics did not significantly reduce serum IS in patients with CKD. The synbiotics did decrease serum PCS. The synbiotics favorably modified the stool microbiome with enrichment of Bifidobacterium and depletion of Ruminococcaceae.	(71)
Bifidobacterium longum in gastro resistant seamless capsule (Bifina) or Bifidobacteria in powder formulation (Lac B)	HD patients were treated with Bifina for 5 weeks, and another group HD patients were treated with Lac B for 5 weeks.	Bifina administration to HD patients is effective in reducing serum IS by correcting the intestinal microflora.	(72)
Synbiotics (Lactobacillus casei strain Shirota and Bifidobacterium breve strain Ya kult as probiotics and galacto-oligosaccharides as prebiotics)	HD patients received synbiotics three times a day for 2 weeks after 2-week pretreatment observation.	Synbiotic treatment resulted in normalization of bowel habits and decrease of serum PCS in HD patients.	(73)
L. casei Zhang (Lac.z)	Individuals with CKD in stages 3–5 (*n* = 62) were underwent randomization to receive either a placebo (*n* = 29) or L. casei Zhang (*n* = 33) as a supplement For 3 months.	Lac.z treatment delayes progression of CKD: did not altered the levels of creatinine, BUN did not altered the safety and tolerability decreased CysC and PTH level The increase in urine albumin-to-creatinine ratio was milder. eGFR decline was much slower.	(45)

Prebiotics is the organic matter that promote the metabolism and proliferation of beneficial bacteria in host. Synbiotics is the combination of probiotics and prebiotics. Prebiotics diets, such as butyrate-releasing high-amylose maize starch diet, were proved to be highly effective for protection against kidney injury (74). Beneficial polyphenols and fructooligosaccharide, as the essential prebiotics in our regular diet, inhibited pathogenic bacteria, and improved inflammation, thus preventing CKD progression (75, 76). In a mouse kidney ischemia/reperfusion model, D-serine, one of the prebiotics, was identified to mitigate AKI via suppressing hypoxia-induced tubular damage and promoting posthypoxic tubular cell proliferation. Meanwhile, D-serine levels in circulation was significantly correlated with renal function decrease in AKI patients (77). Butyrate prevented proteinuria by preserving podocytes of adriamycin nephropathy model in a GPR109a-dependent manner (74). In addition, dietary fiber protected against diabetic nephropathy by SCFAs-mediated activation of GPR43 and GPR109a (78). Synbiotics diets containing prebiotics (glutamine, dietary fiber, and oligosaccharide) and probiotics (Bifidobacterium longum strain) preserved renal function decline and lowed serum IS. Moreover, this kind of synbiotics was proved without serious adverse effects (79). Bupleurum polysaccharides ameliorated diabetic nephropathy induced by STZ (80). Prebiotic–gum acacia (GA) treatment successfully reversed CKD-induced gut dysbiosis and increased butyrate production (81, 82).

In summary, probiotics, prebiotics and synbiotics can be widely applicated to treat kidney diseases by improving gut dysbiosis and metabolic disorders. The species-related selection of these treatments should be dependent on variety of diseases and individuals. Large numbers of rigorous clinical trials are needed to further confirm the effects and safety and optimize the methods and durations of treatment. In addition, bacteria are essentially living organisms and regulated by genes. An interesting research was reported to introduce the targeted genetic manipulation of Bacteroides species in the human gut, which provides a brand-new perspective for gut microbiota interventions to prevent kidney diseases (83).

Different from probiotics treatment to supply several specific bacteria, FMT consists of fecal infusion from healthy donor. So far, the recurrent Clostridium difficile infection has been the only condition on which FMT is accurately efficient with more than 80% of efficiency rate (84). However, no clear evidence supporting a FMT approach in CKD. In CKD mice, FMT decreased PCS accumulation in circulation and improved glucose tolerance, but there was no alteration in renal function (85). FMT and probiotic treatments both targeting on microbiome reconstitution, partly. Suez J et al. found that post antibiotic perturbation, FMT arose a rapid and near-complete recovery within days after administration, while probiotics induced a delayed and persistently incomplete one due to the imperfection of colonization (86). The term and accompanied treatments, especially antibio-therapies, impact on the FMT application. Further studies, especially double-blind, placebo-controlled, are needed to explore the FMT efficiency and adverse events, donor selection and feces management, the timing course and feces amount to ensure that FMT can be a promising option in CKD treatment.

## Conclusions

In this review, we described that gut dysbiosis existed in various kidney diseases, generally manifesting as imbalance between beneficial bacteria and pathogenic bacteria. In many conditions, gut dysbiosis was also accompanied by metabolic disorders, which were the important mediators in gut-kidney axis. Metabolite alterations not only directly impact on kidney disease progression but also indirectly regulate immune responses. Gut-derived immune cells might also migrate to kidney where they served as pathogenic factors. The regulation of gut dysbiosis by probiotics, prebiotics, synbiotics is confirmed to be beneficial to kidney diseases. While the value of FMT remained to be verified.

Nevertheless, there are still several questions to be solved. Firstly, the exact causal relationship between gut dysbiosis and kidney diseases are needed to be confirmed to consider the specific dysbiosis as the target to treat diseases. Secondly, the deeper understanding of the mechanisms of the crosstalk between gut and kidney is needed to explore. Finally, the further clinical trials are needed to confirm the efficiency and safety of probiotics, prebiotics, synbiotics and FMT.

## Author Contributions

CC wrote the manuscript. YY supervised the work and provided financial support. RZ designed the work and provided financial support. All authors contributed to the article and approved the submitted version.

## Funding

This work was supported by the National Natural Science Foundation of China (Grants 81770684, 81974087, 81770681, and 81974086).

## Conflict of Interest

The authors declare that the research was conducted in the absence of any commercial or financial relationships that could be construed as a potential conflict of interest.

## Publisher's Note

All claims expressed in this article are solely those of the authors and do not necessarily represent those of their affiliated organizations, or those of the publisher, the editors and the reviewers. Any product that may be evaluated in this article, or claim that may be made by its manufacturer, is not guaranteed or endorsed by the publisher.
